# Editorial: Next-generation technologies for antibiotic susceptibility testing

**DOI:** 10.3389/fmicb.2026.1845305

**Published:** 2026-05-11

**Authors:** Razi Ahmad, Jun Sung Hong, Jiazhen Chen, Yi-Wei Tang, Santi M. Mandal

**Affiliations:** 1 Indian Institute of Technology Delhi, New Delhi, India; 2 Department of Companion Animal Health and Science, Silla University, Busan, Republic of Korea; 3 Huashan Hospital, Fudan University, Shanghai, China; 4 Chongqing Medical University, Chongqing, China; 5 Department of Biochemistry and Molecular Biophysics, University of California, San Diego, La Jolla, CA, United States

**Keywords:** antimicrobial resistance (AMR), antimicrobial susceptibility testing (AST), clinical implementation, genotype-phenotype correlation, next-generation sequencing (NGS)

The cornerstone of effective antimicrobial therapy, traditional antimicrobial susceptibility testing (AST) remains a double-edged sword. Culture-based methods like disk diffusion and broth microdilution are the gold standard for guiding treatment, yet their inherent inefficiency, requiring 24–72 h for results, directly contributes to the crisis they aim to solve. This delay forces clinicians into empiric, broad-spectrum antibiotic use, a primary driver of antimicrobial resistance (AMR) (Hassall et al., 2024). The collection of articles in this Research Topic captures a field in dynamic flux, collectively demonstrating that the future of AST lies in transcending these limitations through a convergence of novel methodologies, clinical validation, and a deep engagement with practical implementation challenges.

A dominant theme within this Research Topic is the pursuit of rapid, accurate, and scalable AST platforms that bypass the bottlenecks of phenotypic culture. A paradigm shift is evident in the move from indirect genotyping to direct, real-time analysis. For instance, Jakubicek et al. introduce a transformative approach to resistance gene identification using a base calling-free hybrid transformer method for raw nanopore sequencing data. By circumventing the computational and error-prone step of base-calling, this technology accelerates the identification of AMR determinants, exemplifying a powerful trend: the application of deep learning and advanced signal processing to optimize real-time genomics for resistance profiling.

This genomic momentum is further underscored by the work of Li et al., who clinically validate the Xpert Carba-R assay for direct carbapenemase gene detection from respiratory specimens. Their multicentre assessment highlights the tangible impact of nucleic acid–based diagnostics in improving infection management for critical Gram-negative pathogens, where rapid identification of resistance mechanisms is paramount. Complementing these targeted approaches, Aldea et al. provide a comprehensive academic evaluation, mapping the integration of next-generation sequencing (NGS) into clinical microbiology. Their review articulates a future where classical diagnostics are augmented by AI-enhanced procedures and microfluidics, bridging the gap between phenotypic and genotypic paradigms.

Methodological innovation, however, extends beyond the genome. Gómez Estévez et al. introduce ColorPhAST, a rapid visual colorimetric assay that assesses *Escherichia coli* susceptibility to phage therapy. This functional, non-culture-based phenotypic test demonstrates the power of integrating phage biology with simple colorimetric readouts to provide rapid susceptibility patterns. Simultaneously, Cherkaoui et al. show that automated culture-derived platforms, such as PhenoMATRIX^®^ PLUS, remain highly relevant when enhanced with advanced pattern recognition, proving that optimizing existing workflows can yield significant gains in the detection of carbapenemase-producing organisms and other high-priority pathogens.

The wider applicability of these advanced methods is evident in the diagnosis of complex infections. Yang et al. illustrate the power of targeted NGS for diagnosing acute lower respiratory infections, a setting where standard cultures frequently fail. This work underscores that genomic methodologies are not merely for AST but are becoming indispensable tools for comprehensive pathogen detection and characterization. This principle extends to personalized medicine, as highlighted by Gou et al., who investigate the necessity for focused AST to guide customized eradication therapies for *Helicobacter pylori*, a pathogen with globally divergent resistance patterns.

Parallel to these genomic and molecular advances, a renaissance in rapid phenotypic testing is underway. Negin et al. present a novel fluorogenic method for rapid colistin susceptibility testing in *Klebsiella pneumoniae*. This innovation demonstrates that new fluorescence-based readouts can dramatically reduce turnaround times for even the most challenging antibiotics, representing a significant shift toward phenotype-driven, high-sensitivity detection that bypasses lengthy incubation.

Despite this wave of innovation, the path to widespread clinical integration is fraught with persistent challenges that temper enthusiasm. Several contributions in this Research Topic critically address these hurdles. Technological complexity, prohibitive costs, and specialized infrastructure requirements remain formidable barriers, particularly for resource-limited or decentralized settings. Furthermore, the field continues to grapple with a lack of standardized regulatory validation for novel AST systems; clinical trust hinges on demonstrable concordance with established CLSI or EUCAST breakpoints (Cusack et al., 2019). A more profound scientific challenge lies in the genotype-phenotype correlation. Sequencing-based research underscores that the presence of a resistance gene does not always equate to phenotypic resistance, with factors like gene expression, regulatory networks, and epistatic interactions complicating predictions (Eladawy et al., 2025). This necessitates the development of curated databases and sophisticated predictive models that can reliably translate genomic data into robust clinical phenotypes.

Looking forward, the trajectory for next-generation AST is becoming clear. First, the future lies in miniaturization and automation, pushing toward point-of-care (PoC) implementation through lab-on-a-chip devices and integrated optical detectors. Second, the most impactful solutions will likely be multi-modal, combining genotypic, phenotypic, and computational approaches to provide cross-validated, clinically actionable results. Third, establishing rigorous data standards and interoperability frameworks is non-negotiable for the field's success. The precision of genomic AST is only as reliable as the quality of its database curation and the efficacy of consensus interpretation frameworks. Ultimately, even the most revolutionary AST technology will fail to impact patient outcomes without parallel advances in regulatory support, sustainable reimbursement models, and comprehensive training for healthcare professionals.

In conclusion, the articles within this Research Topic collectively paint a picture of a field on the cusp of transformation. From deep learning-powered sequencing analytics to rapid fluorogenic phenotyping, the tools to overcome the limitations of traditional AST are rapidly evolving. The collective enthusiasm and ingenuity on display foster genuine optimism that antimicrobial susceptibility testing is poised to become more rapid, intelligent, and capable of meeting the urgent demands of antimicrobial stewardship in the 21st century. The decisive challenge now lies not in invention alone, but in the concerted effort to standardize, democratize, and seamlessly integrate these powerful new tools into the fabric of global clinical practice.
